# On the crystallography and reversibility of lithium electrodeposits at ultrahigh capacity

**DOI:** 10.1038/s41467-021-26143-9

**Published:** 2021-10-15

**Authors:** Qing Zhao, Yue Deng, Nyalaliska W. Utomo, Jingxu Zheng, Prayag Biswal, Jiefu Yin, Lynden A. Archer

**Affiliations:** 1 Robert Frederick Smith School of Chemical and Biomolecular Engineering, Cornell University, Ithaca, NY 14853 USA; 2 Department of Materials Science and Engineering, Cornell University, Ithaca, NY 14853 USA

**Keywords:** Batteries, Batteries

## Abstract

Lithium metal is a promising anode for energy-dense batteries but is hindered by poor reversibility caused by continuous chemical and electrochemical degradation. Here we find that by increasing the Li plating capacity to high values (*e.g*., 10–50 mAh cm^−2^), Li deposits undergo a morphological transition to produce dense structures, composed of large grains with dominantly (110)_Li_ crystallographic facets. The resultant Li metal electrodes manifest fast kinetics for lithium stripping/plating processes with higher exchange current density, but simultaneously exhibit elevated electrochemical stability towards the electrolyte. Detailed analysis of these findings reveal that parasitic electrochemical reactions are the major reason for poor Li reversibility, and that the degradation rate from parasitic electroreduction of electrolyte components is about an order of magnitude faster than from chemical reactions. The high-capacity Li electrodes provide a straightforward strategy for interrogating the solid electrolyte interphase (SEI) on Li —with unprecedented, high signal to noise. We find that an inorganic rich SEI is formed and is primarily concentrated around the edges of lithium particles. Our findings provide straightforward, but powerful approaches for enhancing the reversibility of Li and for fundamental studies of the interphases formed in liquid and solid-state electrolytes using readily accessible analytical tools.

## Abstract (web summary)

Lithium metal batteries offer high-capacity electrical energy storage but suffer from poor reversibility of the metal anode. Here, the authors report that at very high capacities, lithium deposits as dense structures with a preferred crystallite orientation, yielding highly reversible lithium anodes.

## Introduction

The high theoretical specific capacity (3862 mAh g^−1^, 2062 Ah L^−1^) of lithium metal is rightly cited as the motivation for the recent revival of interest in rechargeable batteries that use Li as the anode. As an element with one of the lowest electronegativity (*χ* = 0.98) and most negative standard electrode potential (*E* = −3.04 V), lithium metal is unsurprisingly highly reactive both under resting and active conditions in an electrochemical cell. Its use as an electrode in closed, high-energy batteries would then appear doomed to failure as all known electrolytes (liquid or solid) will react with lithium metal through chemical and/or electrochemical means to create new materials inside the battery. These transformations consume both electrolyte components and the Li electrode, accelerating battery degradation and failure. Conventionally it is thought that the new materials created by such reactions form a condensed phase, termed the solid electrolyte interphase (SEI) between the lithium metal electrode and electrolyte, which by analogy to what occurs at the graphite anode in Li-ion cells, it is hoped will passivate the Li metal and provide design degrees of freedom for achieving long-term stability of closed battery cells^1–3^. In practice, however, and despite tremendous research, the calendar life of lithium metal batteries remains largely limited by poor reversibility of the anode associated with the consumption of electrolyte components and the Li anode itself.

Identifying and remedying the sources for poor reversibility of Li anodes have  received significant attention from both chemical and physical perspectives. The consensus is that Li’s poor reversibility originates from two major sources^2–4^. First, continuous chemical and electrochemical reactions during closed-circuit charge/discharge operation, as well as during open-circuit storage, will consume both Li and electrolytes. A consequence is that premature failure of practically relevant Li metal batteries (electrolyte-to-capacity ratio <3 g Ah^−1^)^5^, is typically caused by consumption of electrolyte as a consequence of  side reactions with lithium metal. Second, and often simultaneously, uneven Li-plating processes driven by non-uniform ion transport through the new phases formed by electrolyte decomposition products will transform the morphology of lithium into mechanically fragile, mossy and, in some specialized situations, dendrite structures^6,7^. Breakage of these fragile structures  leads to the formation of physical “dead” or “orphaned” lithium in the stripping process owing to the physical isolation under heterogeneous local current^8^. Recent titration gas chromatography studies have revealed that in state-of-art electrolytes (highly concentrated electrolytes, aprotic liquids, and salts with high fluorine content, etc), which in some cases can reach Li plating/stripping reversibility/Columbic efficiency (CE) over 99%, Li loss due to the first of these processes is the main source of the irreversibility of the Li anode^9^. Although the situation concerning dead/orphaned lithium can be resolved through the design of a three-dimensional current collector at the anode^10,11^, under some circumstances non-planar deposition of Li at a heterogeneous SEI can cause run-away Li growth to bridge the battery electrodes, short-circuiting the cells, which raises obvious safety concerns.

As a body-centered cubic metal, (110)_Li_ facets exhibit the lowest migration energy barrier, and are thought less prone to grow into non-planar dendritic morphologies than other crystal facets^12^. Thermodynamic calculations also predict that (110)_Li_ is the most stable Li crystal facet. Li particles are therefore expected to preferentially grow in a rhombic dodecahedron morphology, dominated by the (110)-crystal surface^13^. Thus, under conditions where non-uniform Li transport associated with a heterogeneous SEI can be avoided, dense Li deposits with low porosity and dominantly (110)_Li_ crystallographic facets would be expected. We hypothesized that Li deposition at unconventional (very high, e.g., 20 mAh cm^−2^) capacities in cyclic ether-based electrolytes, unique for their electrochemical stability at highly reducing Li deposition potentials, could open access to a previously unexplored Li deposition regime. Specifically, under high Li deposition capacity, the early growth of lithium electrodeposits should largely be controlled by ion transport in the SEI. Continuous deposition to form high areal capacity and thick structures makes the SEI relatively uniform and facilitates formation of robust ion transport routes, enabling faster interphasial ionic transport. Thereafter, deposition transitions to a second, growth, stage wherein ion transport through the stable thin SEI is not the limiting step. In this stage, we hypothesize that the aforementioned intrinsic thermodynamic effects of lithium will play a dominant role and lithium will preferentially grow as (110)_Li_-dominated structures with more planar morphologies. The result should then favor the creation of highly crystalline Li deposition on a planar electrode with the sought after high reversibility.

**Fig. 1:**
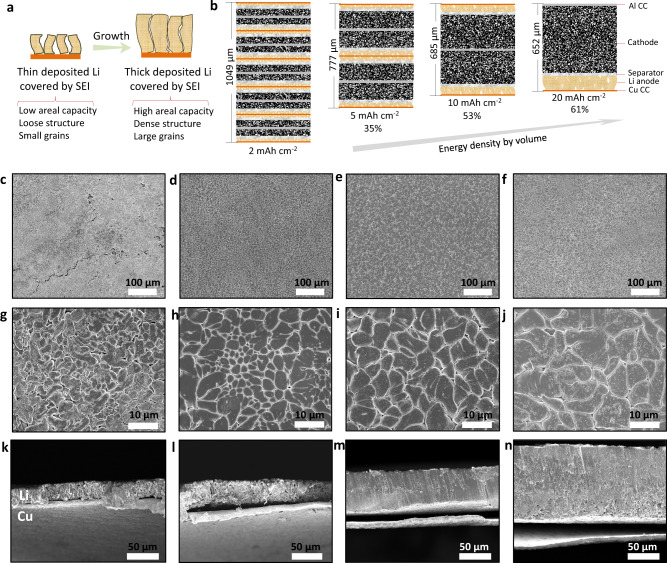
Morphological transition at Li anode at high deposition capacity. **a** Proposed growth mechanism for lithium deposition at electrodes with areal capacity ranging from low to very high. SEI stands for solid electrolyte interphase. **b** Schematic illustrating impact of anode areal capacity on the overall energy density of Li metal full-cell batteries. A high-capacity cathode coupled with dense lithium deposition achieved at 20 mAh cm^−2^ exhibits a 61% increase in volumetric energy density (See details in Methods). CC stands for the current collector. **c**–**n** Morphology evolution of deposited lithium metal from low to high areal capacity. **c**, **g** and **k**, 2 mAh cm^−2^; **d**, **h**, and **i**, 5 mAh cm^−2^; **e**, **i**, and **m** 10 mAh cm^−2^; **f**, **j**, and **n** 20 mAh cm^−2^. **c**–**j** top view; **k**–**n** cross-section view.

According to the above hypothesis, high areal deposition of lithium metal would exhibit denser structures due to the suppression of further reaction between lithium and electrolytes. This is facilitated by the formation of a stable SEI and the limited space in the batteries (Fig. 1a). The continuous growth of lithium through plating also enables formation of bigger grains. The relatively low specific surface area of big particles would be advantageous for a number of reasons, most importantly, the will slow down parasitic chemical and electrochemical reactions, lowering the consumption of electrolytes. As shown in Fig. 1b, when paired with cathodes that can host all of the Li, the Li batteries with thick lithium metal anode exhibit an increase of energy density of the order 61%, from 724 Wh L^−1^ to 1166  Wh L^−1^ (calculated by the operation voltage of 3.8 V). It is important to note that the increase in volumetric energy is not only caused by scaling the areal capacity of the cathode but is also a consequence of the compact/dense structures we report that electrodeposited Li metal forms at high areal capacity. It is understood that at high areal capacity, the cathode may undergo limitation of ion transport; a challenge that will need to be addressed in future works. However, recent works have reported several promising strategies such as segregated nanotube networks (30 mAh cm^−2^)^14^ and non-planar electrode (28 mAh cm^−2^)^15^ towards the creation of cathode architectures to enable faster ion transport at high active material loading.

## Results and discussion

### Dense lithium deposition with large grains at high areal capacity

We herein report on Li deposition in the cyclic ether, 1,3-dioxolane (DOL) over a broad range of conventional and unconventional capacities. The electrolytes used in the study contained a mixture of two salts (2 M lithium bis(fluorosulfonyl)imide (LiFSI) and 0.5 M LiNO_3_) known, respectivelyfor their ability to passivate Li by forming thermodynamically stable fluorine-rich electroreduction products and for strongly coordinating with the ether ring to stabilize DOL against a chemical attack by Li^16^. We find that at high areal deposition capacities Li metal undergoes a transition from high-surface-area loose structures to large, dense structures, with exceptionally high reversibility. The transition is accompanied by the appearance of (110)_Li_ as the most-dominant crystallographic facet.

At a fixed current density of 1 mA cm^−2^ and at high deposition capacity Li deposited on planar Cu substrates manifests a dense, compact morphology composed of µm-sized grains/particles, as confirmed by scanning electron microscopy (SEM). In contrast, the deposits are less compact & mossy, and composed of wire-like structures in commercial carbonate electrolytes (Supplementary Fig. 1). The average surface grain sizes of lithium deposited in the cyclic ether-based electrolyte are also observed to increase uniformly with the increasing of depositing capacities (1.8 μm for 2 mAh, 2.7 μm for 5 mAh, 4.8 μm for 10 mAh, and 6.4 μm for 20 mAh (Supplementary Fig. 2). The thickness can be estimated from the cross-section view of the SEM images to be 22 μm for 2 mAh (Fig. 1k), 35 μm for 5 mAh (Fig. 1l), 56 μm for 10 mAh (Fig. 1m), and 108 μm for 20 mAh (Fig. 1n), respectively. The density of lithium calculated from values evidently range from 44%, 69%, 87%, and 90% of the theoretical bulk Li metal (0.534 g cm^−3^). Our results therefore clearly show that with higher deposit capacity Li electrodeposited in a liquid DOL electrolyte approaches the bulk density of the metal.

### Characteristics of deposited Li with (110)_Li_ dominant crystallographic facets

**Fig. 2:**
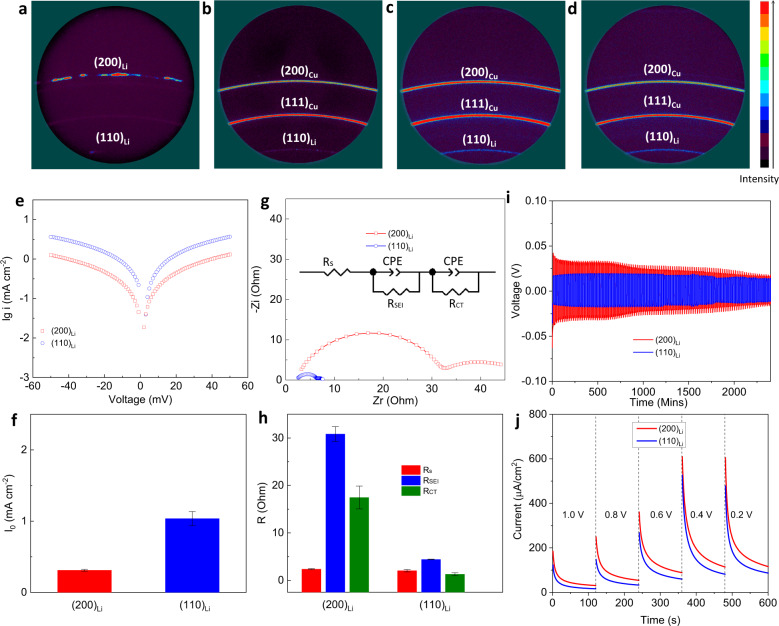
Crystallography, morphology, and kinetics of deposited lithium metal at low, moderate, and high areal capacities. **a**–**d** 2D General area XRD analysis. **a** pristine lithium foil with (200) preferred crystal face orientation. **b**–**d** deposited lithium on Cu substrate with an areal capacity of 5 mAh cm^−2^. **c** 10 mAh cm^−2^, and **d** 20 mAh cm^−2^, showing (110) preferred crystal facet orientation. **e**–**j** kinetic studies of (200)_Li_ and (110)_Li_ electrodes. **e** Tafel plots and **f**, corresponding exchanging current density. The error bars (standard deviation) were calculated based on at least two sets of data. **g**, EIS and **h**, bulk resistance (R_S_), charge transfer resistance (R_CT_), and SEI resistance (R_SEI_) calculated by symmetrical Li||Li electrochemical cells. The error bars (standard deviation) were calculated based on at least two sets of data. **i** Lithium stripping/plating profiles of symmetrical Li||Li electrochemical cells. The current density used for lithium stripping/plating is 1 mA cm^−2^. **j** Chronoamperometry measurements in Li||Cu electrochemical cells. The notation (110)_Li_ is used here to denote deposited Li with a platting capacity of 20 mAh cm^−2^. The notation (200)_Li_ is used to denote Li foil obtained by electrochemically stripping 0.5 mAh cm^−2^ Li at a current density of 0.5 mA cm^2^, conditions where the Li foil can react with the electrolyte.

We investigated the crystal facet orientation of the Li deposits using X-ray powder diffraction (XRD) analysis. Lithium is a BCC metal, and (110)_Li_, (200)_Li_, and (211)_Li_ are normally the dominant crystallographic features observed in XRD patterns. Commercial lithium foil was used as a control and displays a dominant XRD peak corresponding to (200)_Li_, but also exhibits measurable diffraction intensity corresponding to (110)_Li_ (Fig. 2a). In contrast, for lithium plated on Cu foil in the DOL electrolyte, the (110)_Li_ facet are noticeable at moderate capacity (5 mAh cm^−2^), but become dominant at higher Li electrodeposit capacities (20 mAh cm^−2^) (Fig. 2b–d, Supplementary Fig. 3, Supplementary Table 1). It is noted that the presence of the (110)_Li_ facets seem to be an intrinsic characteristic of Li deposition in cyclic ether-based electrolytes; we believe it is a direct reflection of the electrochemical stability of these electrolytes and minimal influence of heterogeneous SEI transport on the deposited Li. We also studied the morphology and structure of lithium metal obtained during the charging process in anode-free Cu|| lithium iron phosphate (LFP) battery cells. At high deposited lithium capacities (10 mAh cm^−2^) on Cu, the results (Supplementary Fig. 4) also show that (110)_Li_ is the dominant crystal facet.

The crystal facets that facilitate lithium stripping/plating with higher exchange current density are expected to result in a lower overpotential, reducing the driving force of electrochemical reactions. We investigated the kinetics of electrochemically deposited lithium-(110)_Li_ in comparison with lithium-(200)_Li_, and characterized the respective exchange current density and charge transfer resistances. Previous thermodynamic studies indicate that (110)_Li_ display faster steady-state nucleation rates than (100)_Li_ facets^17^. As shown in Fig. 2e–f and Supplementary Fig. 5, (110)_Li_ exhibits a markedly higher exchange current density (1.06 mA cm^−2^), in comparison with (200)_Li_ (0.31 mA cm^−2^). Our finding is consistent with a prior experimental report which indicates that strong texturing of (110)_Li_ deposition increases the homogeneity of lithium growth^18^.

Results from electrochemical impedance spectroscopy (EIS) reported in Fig. 2g–h show further that electrodes composed of dominantly (110)_Li_ exhibit much smaller charge transfer (*R*_ct_) and SEI (*R*_SEI_) resistances, in comparison with those with dominantly (200)_Li_. The additional observation is that the bulk resistance for (110)_Li_ and (200)_Li_ are equal, confirming that the other resistances obtained from the equivalent circuit model are reasonable. The faster kinetics at an electrode with dominantly (110)_Li_ is studied further using symmetric lithium electrochemical cells. At a fixed current density, the cells with dominantly (110)_Li_ as electrode show smaller overpotential than the one with dominantly (200)_Li_ electrode (Fig. 2i). The overpotential of the (200)_Li_ symmetrical cells is also seen to become smaller after cycling. Further XRD analysis reveals that this change is caused by the gradual transformation of the Li crystallography, from (200)_Li_-dominant to (110)_Li_-dominant (Supplementary Fig. 6). In comparison, the crystallography of electrodes that were initially (110)_Li_-dominant remains relatively unchanged, even after extended cycling. It should be mentioned here that deposits that produce higher electrochemically active surface area (EASA) would also be expected to facilitate faster electrode kinetics. The EASA is generally known to be a linear function of the non-faradic double-layer capacitance^19^. According to the results in Supplementary Fig. 7, the capacitance of (110)_Li_ (0.26 mF) is indeed higher than (200)_Li_ (0.18 mF), but the difference is not significant enough to explain the large differences in electrode kinetics observed (for example, the exchange current density for (110)_Li_ is 1.06 mA vs 0.31 mA for (200)_Li_). As a first step to understanding the origin of these findings, chronoamperometry was used to empirically evaluate the relative stability of the electrolytes used in the study to different Li crystal facets, by characterizing the current density at various steps voltages above zero. The results reported in Fig. 2j indicate over the complete voltage range studied, the (110)_Li_ exhibits a lower current than (200)_Li_, indicating that less degradation of electrolyte components occur when in contact with the (110)_Li_-dominant electrode.

### Lithium consumption rate at high areal capacity

**Fig. 3:**
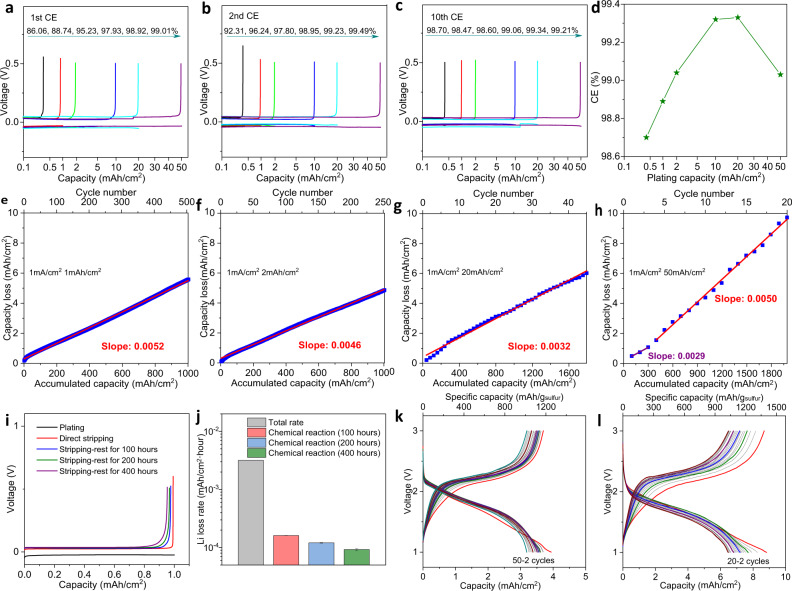
Electrochemical reversibility of lithium as a function of Li plating capacity. **a**–**c** Galvanostatic lithium stripping/plating profiles for Li||Cu electrochemical cells. **a** First cycle, **b** Second cycle, **c** 10th cycle. The Li deposit capacities in **a**–**c** from low to high are 0.4 mAh cm^−2^, 1 mAh cm^−2^, 2 mAh cm^−2^, 10 mAh cm^−2^, 20 mAh cm^−2^, and 50 mAh cm^−2^, respectively. **d** Comparison of average CEs as a function of Li deposit capacity. **e**–**h** Rate of lithium consumption at various plating capacities. **e** 1 mAh cm^−2^, **f** 2 mAh cm^−2^, **g** 20 mAh cm^−2^, and **h** 50 mAh cm^−2^. The bottom *X* axis is the accumulated capacity for both stripping and plating. The top *X* axis reflects the number of lithium stripping/plating cycles. **i** Lithium plating/stripping profiles with or without resting after the plating process. **j** Comparison of total lithium consumption rate and consumption caused by spontaneous chemical reactions. The error bars (standard deviation) were calculated based on at least two sets of data. **k**–**l** Lithium–sulfur battery applications with high areal capacity lithium utilization per cycle (Current density: 0.5 mA cm^−2^). Discharge/charge profiles of lithium–sulfur batteries coupled with high loading sulfur cathode and thin lithium metal anode (**k**, 50 μm, **l** 100 μm). All results from **a** to **j** were obtained at a fixed current density of 1 mA cm^−2^ for both lithium stripping/plating processes. The electrolyte used is 2 M LiFSI in DOL with 0.5 M LiNO_3_.

Our results, therefore, imply that dense lithium deposition at high areal capacity with preferred (110)_Li_ crystal facets should be beneficial for achieving high lithium plating/stripping coulombic efficiencies (CEs). The preferred nucleation of (110)_Li_ crystal facets should also promote faster lithium deposition kinetics and lower reactivity. The two effects are, at least in principle self-reinforcing, as they will result in lower overpotential and hence smaller driven force of electrochemical side reactions, which is consistent with our observations. It is understood, however, that both the Li deposit morphology and intrinsic reactivity of the electrolyte components play different roles. A straightforward way to separate the two effects is to study the reversibility (approximated here using the CE) of Li plating/stripping in Li||Cu electrochemical cells at various deposition capacities. Considering the formation of SEI is the major source of Li consumption, the traditional 3D current collector that can markedly improve the reversibility of lithium metal caused by physical isolation maybe not be an ideal selection to slow down the consumption of anode and electrolytes, which is proved by the results that the CE using 3D Cu foam current collector is even reduced due to anabatic side reactions caused by high-surface-area (Supplementary Fig. 8). Herein, we use commercial 2D Cu foil to study the reversibility of Li deposition. As shown in Fig. 3a, the CE values measured for the first cycle increase with the plating capacity, implying that the consumption of SEI formation is related to the surface area of Li particles. The results show, further, that for any capacity, once a stable SEI is formed during the first cycle, the CE generally increases by the second cycle. The exception is at very high capacities (e.g., 50 mAh cm^2^) where the CE reaches 99.49% (Fig. 3b) in the second cycle, an impressive value at such high capacity, which operates for over 3800 h (43 cycles) with 80% Li retention. Unfortunately, this high CE value is not maintained for the following cycles (Fig. 3c) owing to physical loss (orphaning) of the Li at this extremely high areal capacity (Supplementary Fig. 9). The average CEs capture the competing effects of chemical stability and physical instability. Figure 3d and Supplementary Fig. 10 report that for Li deposit capacities ranging from 0.4, 1, 2, 10, 20, and 50 mAh cm^−2^ the average CE values are, respectively, 98.70%, 98.89%, 99.04%, 99.32%, 99.33%, and 99.03%. Comparing with recent reports, our strategy yields electrodes that exhibit the longest accumulated Li plating/stripping capacity and among the highest CEs reported in the literature (Supplementary Table 2)^20–29^. For completeness and in order to assess the general applicability of our approach, we also evaluated CEs in galvanostatic cycling experiments using a carbonate electrolyte (10 m LiFSI in DMC). The average CE is reported to increase from ~96.4% to 97.8% after increasing the plating capacity from 0.5 mAh cm^−2^ to 5 mAh cm^−2^ (Supplementary Fig. S11).

The details of the Li capacity loss either by electrochemical or chemical reactions are of interest and analyzed in Fig. 3e–h. In general, we find that the apparent Li consumption rate is a decreasing function of the accumulated capacity in the lithium stripping/plating experiment. For Li plating capacities below 20 mAh cm^−2^, we calculated the slope through linear fits over the full range of cycles studied. Increasing the areal capacity from 1 mAh cm^−2^ to 20 mAh cm^−2^, the consumption rate is reduced from 5.2 × 10^−3^ (unit: mAh per hour) to 3.2 × 10^−3^. For a high plating capacity of 50 mAh cm^−2^, we found the slope for the first three cycles is noticeably different from those obtained in the subsequent cycle, which we attribute to the accumulation of morphological effects for long cycles. We have therefore separated the cycling into two regions (1–3 cycles, 4–20 cycles) to calculate the values of the slope reported. Increasing the areal capacity up to 50 mAh cm^−2^ can further decrease the consumption rate to 2.9 × 10^−3^, but as noted earlier this benefit is short-lived due to metal orphaning at the high capacities.

The consumption of Li in forming the SEI can occur either by electrochemical reactions or spontaneous chemical reactions of electrolyte components with the deposited Li. Previous studies have suggested that Li can also be consumed by spontaneous corrosion processes involving a Kirkendall-type mechanism^30^. Li metal loses can reach over 2–3% after aging only 24 h^31^. Our studies in Fig. 3i–j show that chemical reactions play a relatively minor role in the SEI formation process in cycle ether-based electrolytes. A capacity loss of 1.67 × 10^−4^ mAh per hour is found in a 100 h test and 1.21 × 10^−4^ mAh per hour in a 200 h test; taking up <5% of total loss. We find, instead, that the consumption of Li is primarily driven by electrochemical reactions and that hypothesize that the effect should be exacerbated at higher current density because the overpotential is higher. The hypothesis is confirmed in galvanostatic CE experiments in Li||Cu electrochemical cells under various current densities (Supplementary Fig. 12). The results show that the rate of capacity loss rises from is 4.3 × 10^−3^ mAh per hour at lower current density (0.5 mA cm^−2^), and consecutively increases to 5.4 × 10^−3^, and 6.1 × 10^−3^ as current density increases from 2 mA cm^−2^ to 5 mA cm^−2^, respectively. It should be noted here that high current density can also lead to physical isolation and thus accelerate the consumption of lithium metal^32^.

The cycle ether electrolyte with LiNO_3_ addition is found to exhibit stability over thousand cycles for sulfur cathode at low areal capacity <0.5 mAh cm^−2^ (N:P = 30:1) (Supplementary Fig. 13). As an assessment of the practical relevance of our findings, we created Li-S batteries composed of high areal capacity lithium utilization per cycle with a low anode-to-cathode capacity ratio (N:P = 2:1) and investigated their galvanostatic cycling behaviors. The Li-S batteries used sulfur/PAN (polyacrylonitrile) nanocomposites with designed nominal areal capacity either 5 mAh cm^−2^ or 10 mAh cm^−2^ are applied as the cathode (estimated based on the theoretical specific capacity of sulfur)^33^. The results reported in Fig. 3k–l support the promise of the design. Both batteries can operate with similar discharge/charge profiles. The capacity retention is 81% after 50 cycles for 5 mAh cm^−2^ cathode, and 75% after 20 cycles for 10 mAh cm^−2^ cathode. The capacity fading is known to be caused by the formation and dissolution of polysulfides in ether-based electrolytes, and by the consumption of electrolytes due to parasitic chemical and electrochemical reactions with lithium. We found few Li-S coin cells have been operated under rigorous conditions (anode to cathode ratio is ~2). Previous reports on Li-S pouch cell with thin lithium anode of 50 μm, the electrolyte of 1 M LiTFSI + 0.3 M LiNO_3_/DOL-DME and sulfur cathode with the practical capacity of ~3.6 mAh cm^−2^, display the capacity retention of ~50% after 130 cycles^34^. In comparison, the capacity retention for Li-S batteries in this work is 51% after 140 cycles (Supplementary Fig. 14). It is important also to note that for the purpose of this initial demonstration that we intentionally keep focused on the Li anode design, we made no efforts to manipulate design the cathode structure or the electrolyte-to-cathode ratio, both of which are known in the literature to be important in achieving long cycle life. Anode-free Cu||LFP batteries with high areal capacity are also designed to demonstrate the advantages of high areal electrodes. As results shown in Supplementary Figs. 15 and 16, as the areal capacity increases, there is a clear trend towards longer operation time at the same areal current density.

### A straightforward strategy for interrogating the SEI

Our results suggest that the SEI on Li is formed primarily by electroreduction of electrolyte components. It is also the major source of lithium loss at moderate capacities; the structure and composition of the SEI are therefore of considerable interest. By means of the focused ion beam cyro-TEM, recent work has shown that both the chemical composition and nanoscale structure of the SEI formed on pristine Li in carbonate ester-based electrolytes are complex^35^. Our finding that highly reversible Li stripping/plating can be achieved at high areal capacity in ether-based electrolytes raises the perhaps obvious question about the nature of the SEI formed in this case. Here, we take advantage of the fact that at the high areal capacities studied the extent of electroreduction reactions of electrolyte components at the Li electrode would be greater, meaning that the signal to noise associated with the more substantial SEI would be higher, which would make it easier to interrogate using less-specialized surface analytical chemistry techniques.

**Fig. 4:**
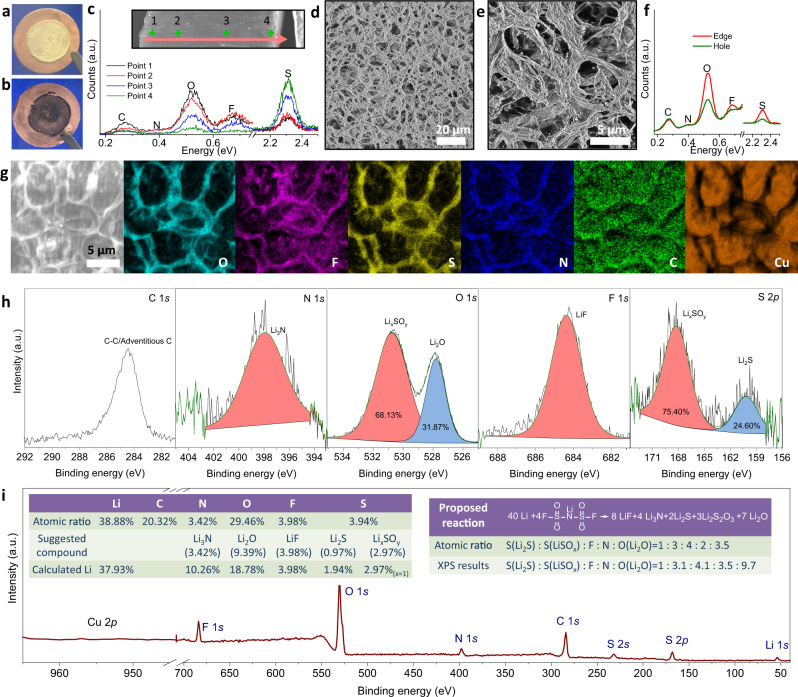
Morphology and composition of the solid electrolyte interphase (SEI) formed on Li. Digital photos of **a** deposited lithium (20 mAh cm^−2^) on Cu, and **b** material that remains after stripping lithium from Cu. **c** EDX spectra taken from the cross-section of deposited lithium from outer to inner edge (Marked points in the inset SEM image). **d**, **e** SEM image of the porous SEI left on Cu foil after stripping away the deposited Li. **f** Corresponding EDX spectra of SEI either on the edge or in the center of the pores of the residual porous material. **g** SEM-EDX O, F, S, C, N, Cu element mapping of SEI. **h** High-resolution XPS analysis of C *1* *s*, N *1* *s*, O *1* *s*, F *1* *s*, S *2p*. **i** Atomic ratio analysis from XPS survey spectra with inserted supposed compositions and SEI formations process.

To interrogate the SEI formed on Li in the ether electrolyte we employed the following two-step procedure. In the first step, 20 mAh cm^−2^ lithium is deposited on a Cu current collector in a Li||Cu electrochemical cells. As shown in Fig. 4a the process leads to the formation of a dense Li metal layer on the Cu substrate. In the second step, the voltage is elevated to 0.5 V at a constant current density to strip all (as evidenced by an increase in the voltage) electrochemically reactive lithium from the Cu. Figure 4b shows that a visible and substantially black material remains on the Cu foil after the Li is stripped. This method is advantageous because it provides insights into the physical and chemical characteristics of the interphase formed on Li at potentials below 0 V (lithium plating process). Energy-dispersive X-ray (EDX) spectroscopy analysis across the deposited Li (Fig. 4c) reveal that surface is enriched in elements O, F, and S. The S signal is strongest at the bottom, and the intensity gradually reduces from the bottom to the top, suggesting the reaction kinetics of S species (Li_2_S, Li_x_SO_y_ etc) is faster than other species. In comparison, O and F signals in general exhibit the reverse tendency, indicating that these interfacial species (Li_2_O, LiF etc) are endowed with slower kinetics than S and prefer to generate on the out layer of deposited Li. SEM analysis of the black material formed after stripping the Li deposits from the Cu current collector reveals an obvious, porous morphology (Fig. 4d, e). The size of the pores (Supplementary Fig. 17, average 5.9 μm) is consistent with the size of lithium particles (average 6.4 μm), indicating that material, which we now designate the SEI, appears to serve as a connector between the Li particles. EDX point spectra (Fig. 4f) of the porous material again reveal that C, N, O, F, and S are the dominant elements in the SEI. SEM-EDX mapping provides a more-detailed analysis (Fig. 4g) of the spatial distribution of the respective elements. The distribution of O, F, S, and N are consistent with the shape of the porous SEI, indicating that these elements are likely components in the same material. However, the spatial distribution of C is not consistent with the shape of the SEI, suggesting that the carbon source is different (e.g., electrolyte residue, adventitious carbon from the environment, etc.). Meanwhile, the strong Cu signal in the hole of pores and weak Cu signals on the edge of pores indicates that Cu likely does not participate in the formation of SEI.

XPS analysis provides complementary information that can be used to obtain additional insights about the detailed chemical composition of the SEI. The C 1 *s* carbon spectra display a typical peak that can be conclusively assigned to adventitious carbon. This indicates that in the cyclic ether electrolyte, inorganic compounds are the major remaining species after extraction of Li deposited on the Cu substrate (Fig. 4h). N 1 *s*, O 1 *s*, F 1 *s,* and S 2*p* high-resolution spectra confirm that the inorganic compounds are mainly Li_3_N^36^, Li_2_O^37^, LiF^38^, Li_2_S, and Li_x_SO_y_^38^. Analysis of the XPS survey spectra reveals further that the atomic ratio of Li is 38.88%, which is viewed in tandem with the above inorganic compounds. We propose that the decomposition of the salts (LiFSI) on the surface of lithium has a dominant role in the formation of the SEI. Specifically, the ratio of Li_2_S, LiSO_x_ (suggested as 0.5 Li_2_S_2_O_3_) and LiF is consistent with the proposed SEI formation reaction presented in Fig. 4i. We note however that the atomic percentages of Li_3_N and Li_2_O deduced from this reaction are lower than what is observed, which can be rationalized in terms of decomposition of LiNO_3_, and the inevitable oxidation of Li by residual oxidation or solvent, which can also generate Li_2_O and Li_3_N^36^. The components of SEI are further studied using attenuated total reflectance-Fourier transform infrared spectroscopy (ATR-FTIR). Significantly, no peaks associated with the C-H vibration are apparent at the interphase. Vibrations associated with SO, Li_2_O are detected in the SEI^37,39^, consistent with the finding from XPS analysis that the SEI formed on Li in a DOL electrolyte is primarily inorganic (Supplementary Fig. 18a). Taken together, these results challenge the conventional view that DOL protects Li by polymerizing on the surface of lithium metal to form a polymeric SEI, which is thought to provide the interfacial elasticity needed to enable higher electrode reversibility^7,40^. Detailed comparisons of electrolytes before and after electrochemical cycling suggest that in addition to the insoluble inorganic-rich SEI formed on Cu, there is also an organic component that may be soluble in the electrolyte and can be detected on the separator (Extended Data, Supplementary Fig. 18b); this component appears to be highly dynamic, and not located in the structural SEI that remains after Li stripping.

To evaluate this last point, we characterized Li reversibility at Cu substrates with a pre-formed SEI created when a high-capacity Li is first plated and stripped from Cu. The results reported in Supplementary Fig. 19 show that the CE for such a substrate increase from 88.74% to 95.56% for the first cycle, a large increase, but still remains below 99%. Introducing the organic dynamic SEI into the cell design yields a higher initial CE of 98.03% and the average CE for 1 mAh cm^−2^ also increases from 98.89% to 99.08% (Supplementary Fig. 19b). With the SEI protection, the reversibility of Li in commercial carbonate electrolytes also improves notably (Supplementary Fig. 19d).

In summary, we demonstrate that increasing the plating capacity of lithium results in dense structures of metal deposition with large grains. Lithium plating with a capacity of up to 20 mAh cm^−2^ displays the space utilization of 90% and CE of 99.33%. Further study on crystal structures unveils that the plating lithium in high areal capacity is highly orientated in (110) crystal face, which is further proved to display fast kinetics of lithium stripping/plating with higher exchange current density but simultaneously exhibit elevated stability towards electrolyte. Benefiting from the thick Li deposition, the morphology of SEI has been able to investigate through a more visible approach. It was found that inorganic-rich SEI is generated tightly around the edges of lithium particles. We note that a handful of electrolytes have been reported that show even higher CEs at low plating capacity and better oxidation stability than the ones studied here^41^. However, the performance of these electrolytes at high plating capacity is unknown. We believe the results reported here will encourage more approaches to reassess these electrolytes. The upper limit of battery energy densities will have a breakthrough with the design of thick Li anode.

## Methods

### Energy density calculation

The energy density is calculated by anode-free pouch cells in an ideal charge state with a total areal capacity of 20 mAh cm^−2^. The thickness of lithium anode is applied according to the SEM analysis, which is 22 μm for 2 mAh cm^−2^, 35 μm for 5 mAh cm^−2^, 56 μm for 10 mAh cm^−2^, and 108 μm for 20 mAh cm^−2^. The thickness of the cathode is estimated as 50 μm for 2 mAh cm^−2^, 125 μm for 5 mAh cm^−2^, 250 μm for 10 mAh cm^−2^, and 500 μm for 20 mAh cm^−2^. The thickness of Cu current collector that is used to load lithium is 9 μm. The thickness of Al current collector that is used to load the cathode is 15 μm. The thickness of separator soaking with electrolyte is 20 μm. When considering the assembly of batteries, 10 layers of cathodes (five pieces, double sides) need to be in parallel together and will deposit 10 layers of lithium on Cu substrate with the same areal capacity of 2 mAh cm^−2^ in the charging process. In comparison, one layer of the thick cathode (one-piece, single side) can be coupled with one layer of Cu substrate that will deposit lithium with an areal capacity of 20 mAh cm^−2^ in a full charge state. Owing to both the dense lithium deposition with higher space utilization at higher areal capacity and the simplification of separators/current collectors, thick Li deposition remarkably reduces the thickness of batteries. The thickness of pouch cells excluding packages for 2 mAh cm^−2^, 5 mAh cm^−2^, 10 mAh cm^−2^, and 20 mAh cm^−2^ deposited Li are 1049 μm, 777 μm, 685 μm, and 632 μm, respectively. If the working potential of batteries is estimated as 3.8 V (NCM811 cathode), the corresponding energy densities for the above pouch cells are 724 Wh L^−1^, 978 Wh L^−1^, 1109 Wh L^−1^, and 1166 Wh L^−1^, respectively. The high-capacity strategies are also applicable for other batteries that applying Li metal anode such as lithium–sulfur batteries, in which the lithium will be re-deposited on the anode in the charging process.

### Electrolyte preparation

Electrolytes were prepared in the Ar-filled glovebox (Inert Inc.) with the content of O_2_ and H_2_O lower than 0.5 ppm. LiFSI was purchased from Oakwood Products Inc. (99% purity). Lithium nitrate (LiNO_3_) is purchased from Chem-Impex Int’l. Inc. (99.99% metal basis). All salts were used without further purification. DOL solvent is purchased from Sigma-Aldrich (99.8%). Several pieces of lithium foils were put into DOL solvent to chemically “dry” the solvent before usage.

### Electrochemical cells test

Coin 2032 cells were assembled in glovebox for the electrochemical studies. For electrochemical Li||Cu cells, lithium foil was cut into a round shape with a diameter of 3/8 inch and acted as anode. Two pieces of Al_2_O_3_ coated celgard membrane was used as a separator. No low current density formation process was applied for all electrochemical Li||Cu cells. The electrolyte amount of electrochemical cells was ~60–70 μl. Cu foil with the size of 5/8 inch was firstly washed with dilute HCl (0.1 M) through the sonication method and then rinsed with water. After rinsing, the Cu foils were quickly transferred to a vacuum oven and dried overnight at 50 °C before assembling batteries. LFP cathodes were sandwiched with two pieces of carbon cloth^14^, and were used to assemble anode-free Cu||LFP batteries. Li||S batteries were prepared with thin Li (50 μm or 100 μm) as anode, and PAN-S composite as cathode. PAN-S compose was prepared through heat treatment of PAN and sulfur at 450 °C^32^. The PAN-S cathode was prepared with a weight ratio 70 wt% PAN-S, 15 wt% Ketjen black carbon, 15 wt% LITHion^TM^ binder (Ion Power), and sandwiched by two pieces of carbon cloth.

### Materials characterizations

SEM images and EDX mapping analysis were obtained on the instrument of Leo 1550 Field Emission SEM carried with Bruker EDX detector. Rigaku X-Ray diffractometer and Bruker D8 General Area Detector Diffraction System were used to take XRD and 2D-XRD patterns, respectively. ATR-FTIR analysis was conducted on Thermo Scientific FTIR spectra. The chemistry information of SEI was tested on X-ray photoelectron spectroscopy SSX-100 (XPS). Galvanostatic lithium plating/plating and discharge/charge measurements were performed on Neware battery tester at room temperature. EIS was performed on Solartron from a frequency range from 50 HZ to 1 HZ at AC polarization of 10 mV. Cyclic voltammetry (used for characterizing the exchange current density) and Chronoamperometry test were performed on a CH 600E electrochemical workstation.

### Deposited lithium studies

The deposited lithium on Cu was collected from electrochemical Li||Cu cells through the Galvanostatic lithium plating process. After disassembling the cells inside the glovebox, the lithium on Cu was washed by pure DOL solvent and naturally dried in glovebox before characterizations. For XRD characterizations, the deposited lithium on Cu with an areal capacity of 5 mAh cm^−2^, 10 mAh cm^−2^, and 20 mAh cm^−2^ was collected from Li||Cu cells after galvanostatic lithium plating at a current density of 1 mA cm^−2^. After washing and dring in the glove box,  the surface of deposited samples was fully covered and sealed with parafilm inside the glovebox. The pristine Li foil was also protected using the same method. All the samples collected in this manner were stored in an argon-filled glass vial before XRD characterization. Through this approach, we have observed that it is possible to limit moisture/air exposure and for Li to retain its metallic luster, before and after XRD measurements.

### Lithium consumption caused by spontaneous chemical reactions

Li||Cu electrochemical cells were firstly cycled at 1 mA cm^−2^ with a plating capacity of 1 mAh cm^−2^ over 50 cycles. Then on the following cycle, after 1 mAh cm^−2^ Li was plated on Cu, the cells were rested for 100 h to 600 h before stripping. Owing to the chemical reaction between lithium and electrolytes, a partial amount of lithium will become inactive after resting.

### SEI formed on Cu

In order to study the SEI formed on Cu substrate, 20 mAh/cm^2^ lithium was plated on Cu using electrochemical Li||Cu cells, followed by a stripping process until the voltage reached 0.5 V. The cells were disassembled in a glovebox, and Cu foils were washed using pure DOL (purified by lithium metal) and convectively dried in the glovebox. For XPS analysis, an inert transfer chamber was used to load the samples and directly transferred them to the testing machine without contacting air. For SEM and EDX analysis, the SEM holder was first used to load samples in the glovebox. Then the holder was sealed and filled with argon in the glovebox. Finally, the holder was quickly transferred to SEM. The transfer process was <5  s to avoid exposing sensitive interphase to the air.

## Supplementary information

Supplementary Information

## Data Availability

All datasets generated and analyzed during the current study are available from the corresponding author (LAA) on reasonable request.
