# Supplementary material for: On the crystallography and reversibility of lithium electrodeposits at ultrahigh capacity
Source: Nat Commun. 2021 Oct 15;12:6034. doi: 10.1038/s41467-021-26143-9 (PMC8519946; doi:10.1038/s41467-021-26143-9)
Supplement: Supplementary file 1 — Supplementary Information [file 41467_2021_26143_MOESM1_ESM.pdf]

Supplementary information

On the crystallography and reversibility of lithium electrodeposits at ultrahigh capacity

Qing Zhao[1], Yue Deng[2], Nyalaliska W. Utomo [1], Jingxu Zheng[2], Prayag Biswal [1], Jiefu Yin[1], Lynden Archer[1,2]*

[1]Robert Frederick Smith School of Chemical and Biomolecular Engineering, Cornell University, Ithaca, NY, 14853, USA.

[2]Department of Materials Science and Engineering, Cornell University, Ithaca, NY, 14853, USA.

*laa25@cornell.edu.

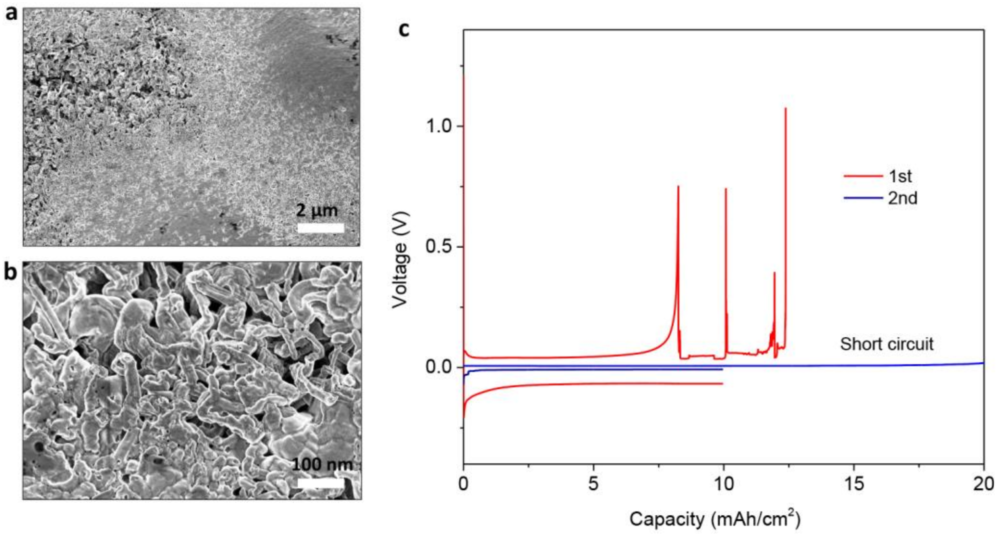

Supplementary Figure 1 | Instability of high areal capacity lithium deposition in carbonate electrolyte. a-b, SEM images of deposited Li on Cu substrate. **c**, Galvanostatic lithium stripping/plating profiles for Li||Cu electrochemical cells. The electrolyte is 1M $LiPF_6$ in ethylene carbonate (EC)/dimethyl carbonate (DMC) (1:1 by volume). The current density is 1mA cm^{-2}.

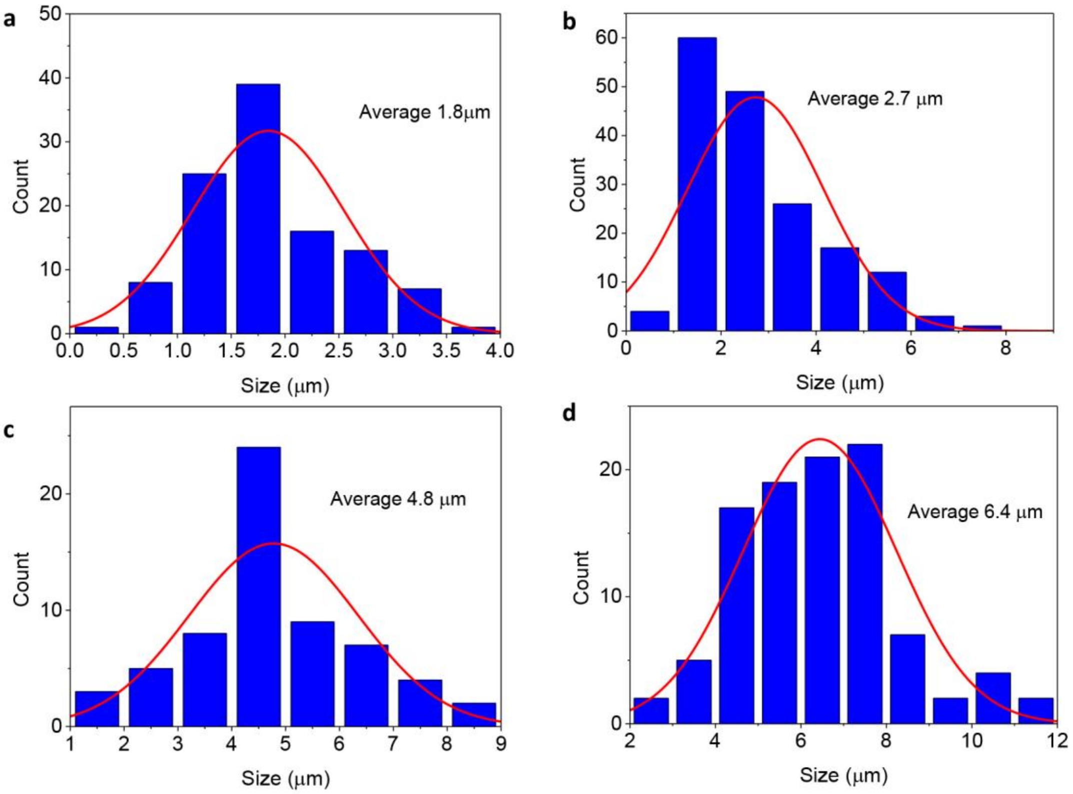

Supplementary Figure 2 | Size distributions of grain diameter of deposited lithium metal on Cu substrate. Grain diameter of deposited lithium with areal capacity of **a**, 2 mAh cm^{-2}, **b**, 5 mAh cm^{-2}, **c**, 10 mAh cm^{-2}, **d**, 20 mAh cm^{-2}.

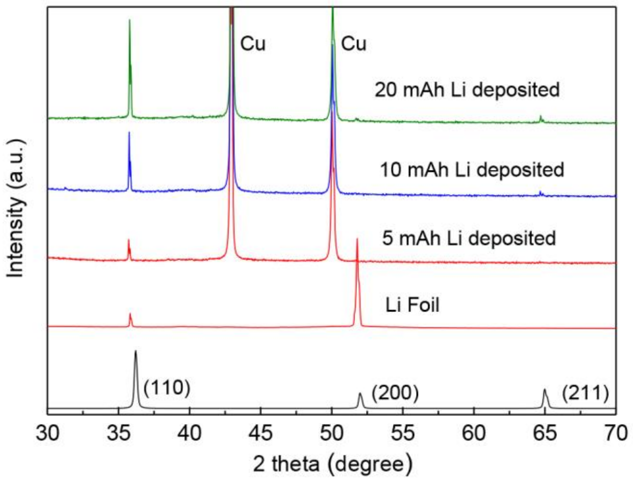

Supplementary Figure 3 | XRD patterns of deposited lithium on Cu foil with different capacities. Black line is the standard XRD pattern of Li.

Supplementary Table 1 | Relative intensity of various crystal facets calculated through XRD patterns.

	Relative intensity (%)		
	**(110)**	**(200)**	**(211)**
**Li foil**	1.00 (12.9%)	6.73 (87.1%)	--
**5 mAh Li deposited**	1.00 (88.3%)	0.061 (6.4%)	0.071 (6.3%)
**10 mAh Li deposited**	1.00 (90.4%)	0.027 (2.4%)	0.079 (7.1%)
**20 mAh Li deposited**	1.00 (91.2%)	0.025 (2.3%)	0.072 (6.6%)

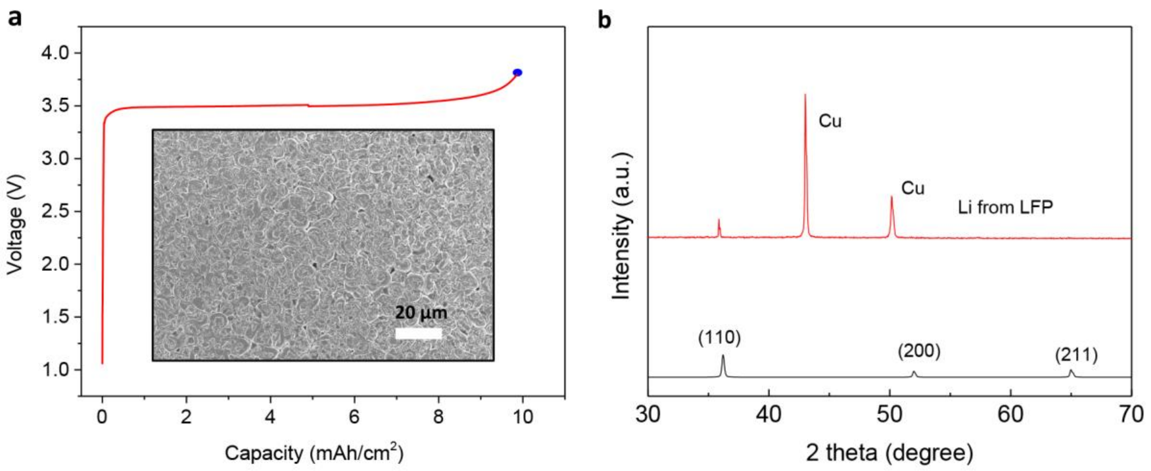

Supplementary Figure 4 | Structure and morphology evolutions of lithium metal achieved from Cu|| LFP anode free batteries. a, Charge profiles of the batteries at the current density of 1mA cm^{-2}. Inset, SEM image of Li obtained from marked blue point. **b**, XRD pattern of lithium derived for LFP. Black line is the standard XRD patterns of Li.

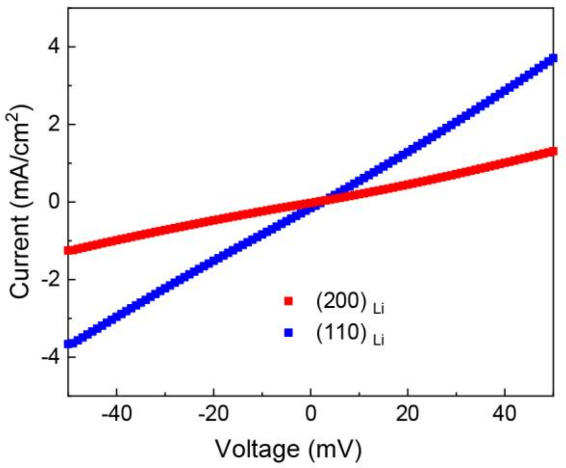

Supplementary Figure 5 | IV curves of symmetric Li batteries with $(200)_{Li}$ and $(110)_{Li}$ electrode. The sweep rate is 2 mV s^{-1} with the voltage range from -50 mV to 50 mV.

Note: The exchange current density was calculated through fitting the linear region of Tafel plots (from 50 mV to 30 mV, and -50mV to -30 mV). Tafel plots were obtained by Logarithm current density of I-V curves.

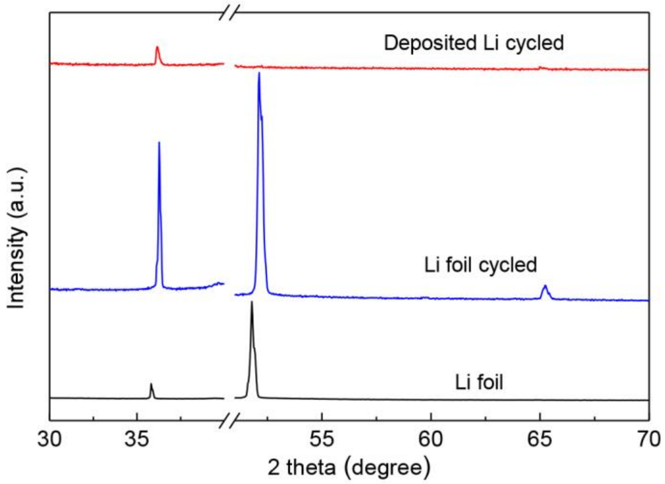

Supplementary Figure 6 | XRD patterns of lithium metal during stripping and plating process.

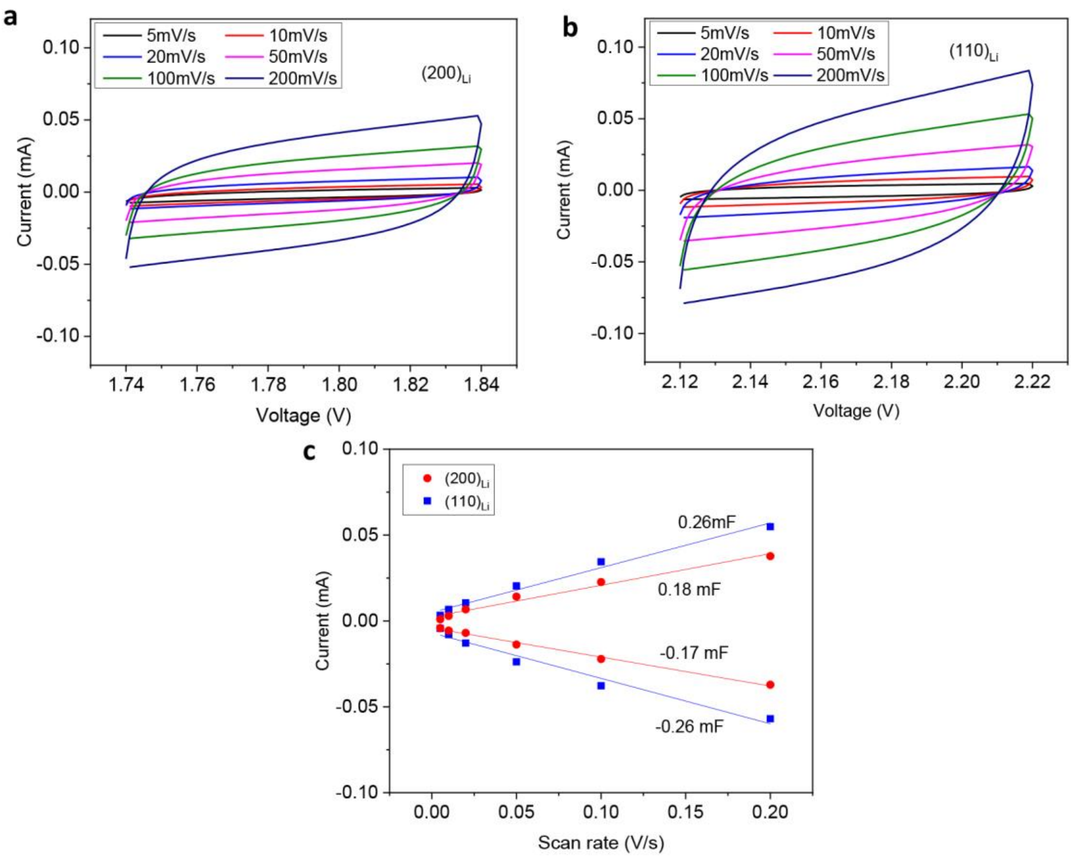

Supplementary Figure 7 | Double-layer capacitance measurements of electrochemical Li||Cu cells. Cyclic voltammograms of non-Faradaic region at different scan rate. **a,** $(200)_{Li}$||Cu and **b,** $(110)_{Li}$||Cu electrochemical cells. **c,** The cathodic/anodic currents and linear fit with scan rate.

Note: Double-layer capacitance is obtained through CV measurement in the voltage range where no apparent Faradaic processes take place.[1] In our experiment, the voltage range is 0.1 V centered at the open-circuit voltage (OCV).

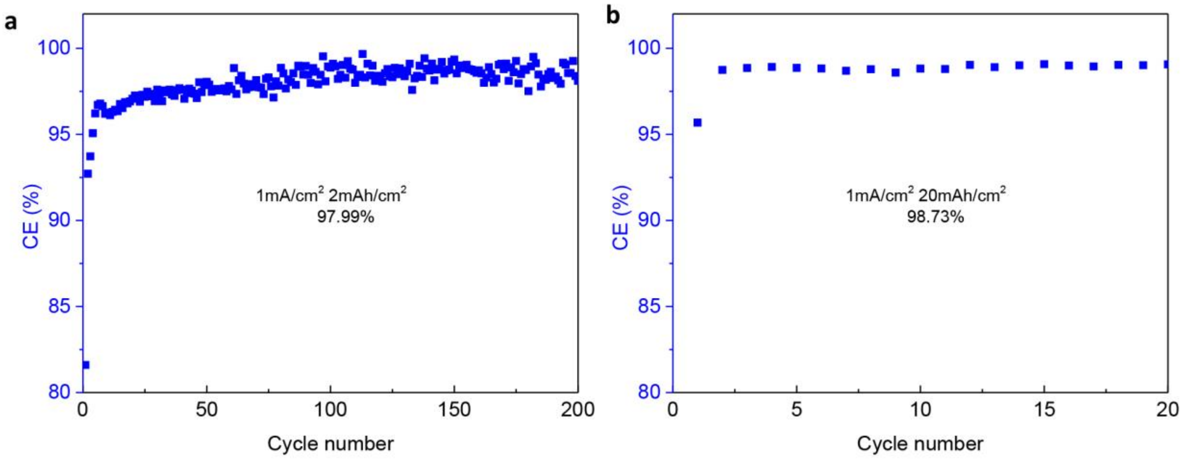

Supplementary Figure 8 | Coulombic efficiencies (CEs) of Li||3-D Cu foam electrochemical cells with different plating capacity. **a**, 2 mAh cm^{-2}, **b**, 20 mAh cm^{-2}. The current density of lithium stripping/plating is 1mA cm^{-2}.

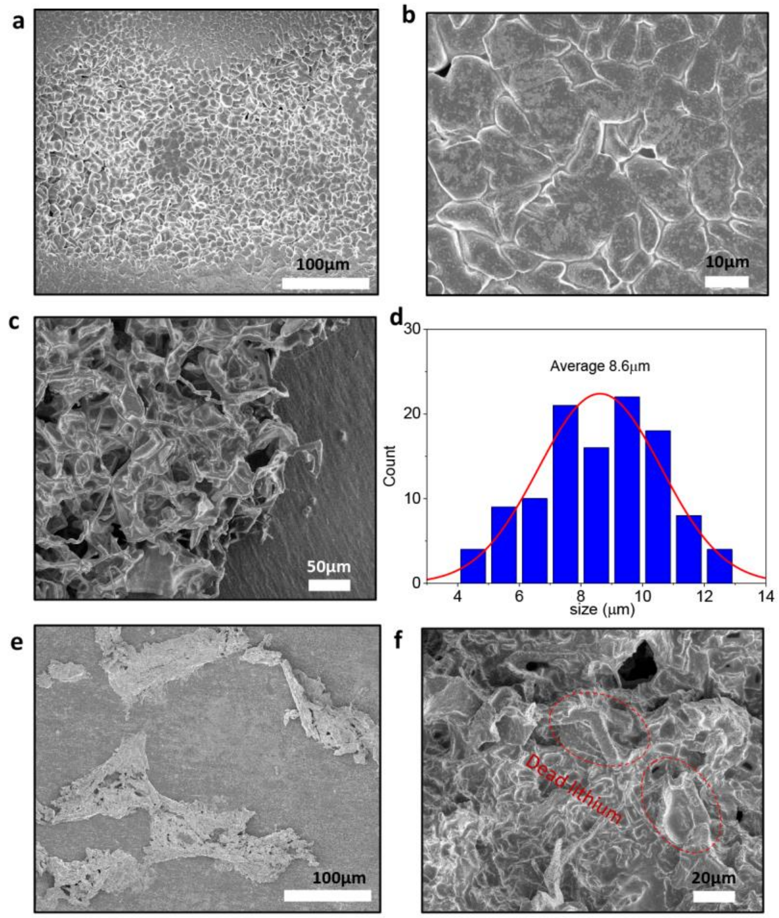

Supplementary Figure 9 | Morphology of lithium stripping and plating at extremely high areal capacity (50 mAh cm^{-2}). a-c, SEM images of deposited Li on Cu substrate. **d**, Average size distribution of deposited Li. **e-f**, Morphology of dead lithium on Cu after stripping process.

Note: When the deposited capacity of lithium increases to 50 mAh cm^{-2}, the surface of deposited lithium is not as dense as at 20 mAh cm^{-2}. The central parts of deposited lithium are relative compact with average grain size of 8.6 μm. Edge effects become very obvious with emerging of wire-like Li due to uneven pressure inside the coin cells. On charging (stripping) process, parts of lithium are irreversible. We conclude that the reduction in CE during further cycles for very thick films is due to the physical “orphaning” of Li at these thicknesses, which is also confirmed by the shaking CE under high plating capacity (Supplementary Figure 10f).

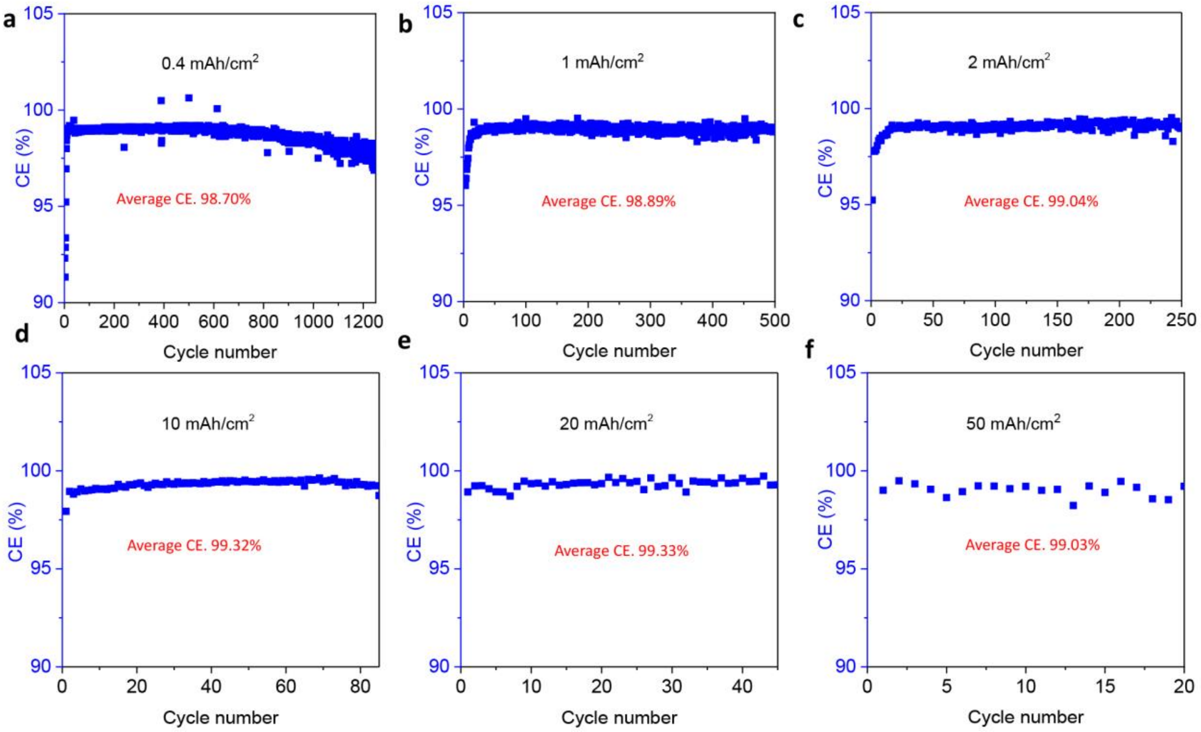

Supplementary Figure 10 | Coulombic efficiencies (CEs) of Li||Cu electrochemical cells with different plating capacity. **a**, 0.4 mAh cm^{-2}, **b**, 1 mAh cm^{-2}, **c**, 2 mAh cm^{-2}, **d**, 10 mAh cm^{-2}, **e**, 20 mAh cm^{-2}, and **f**, 50 mAh cm^{-2}. The current density of lithium stripping/plating is 1mA cm^{-2}.

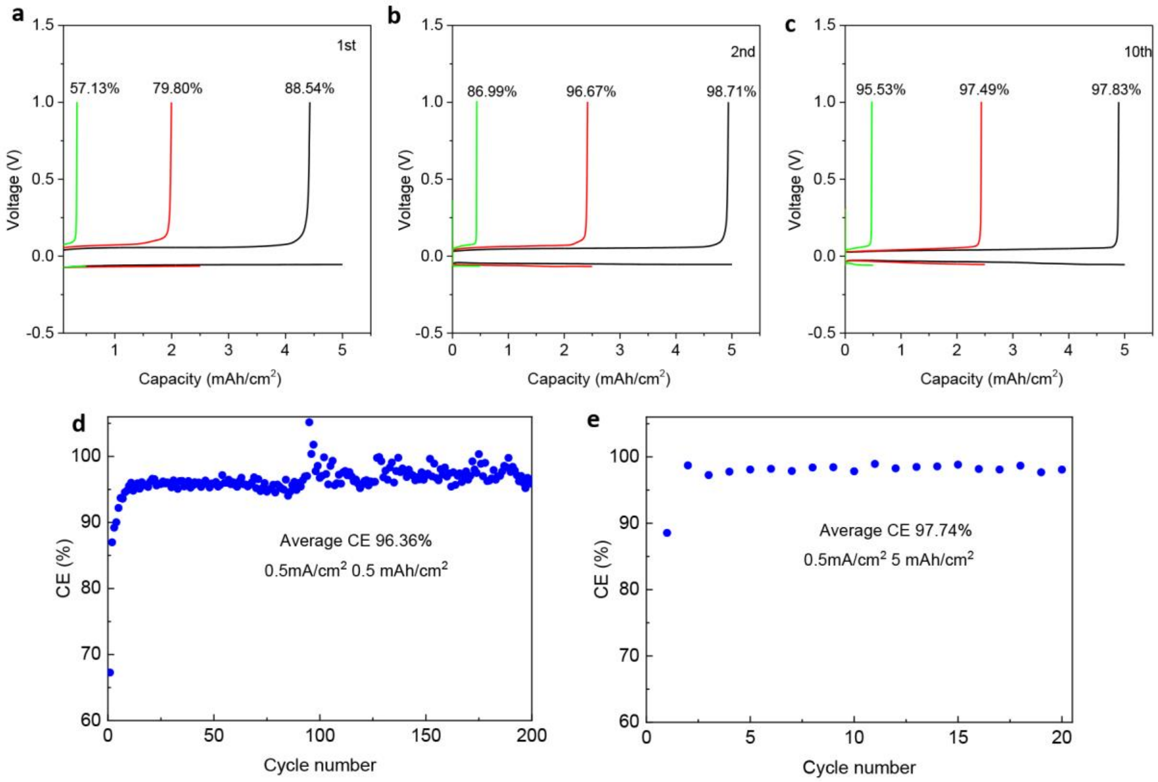

Supplementary Figure 11 | Extend applications of higher CEs with increasing plating capacity of lithium in carbonate electrolytes. Galvanostatic lithium stripping/plating profiles for Li||Cu electrochemical cells. **a,** First cycle, **b,** Second cycle, **c,** 10th cycle. Corresponding long-term CEs of Li||Cu electrochemical cells with plating capacity of **d,** 0.5 mAh cm^{-2} and **e,** 5 mAh cm^{-2}**.** The current density for lithium stripping/plating is 0.5 mA cm^{-2}. The electrolyte is 10m LiFSI in DMC.

Supplementary Table 2 | Comparisons of electrochemical Li||Cu cells with state-of-the-art electrolyte

**Electrolytes**	**Current density / areal capacity (average CE)**	**Accumulated capacity (Stripping +plating)**	**Ref**
1.3 m LiTFSI + ~0.04 m $LiNO_3$ / SL-HFE	0.5 mA cm^{-2} / 1 mAh cm^{-2} (99.0%)	~0.32 Ah cm^{-2}	2
10 M LiFSI / DMC<br>10 M LiFSI / EC-DMC	0.2 mA cm^{-2} / 1 mAh cm^{-2} (99.2%)<br>0.2 mA cm^{-2} / 1 mAh cm^{-2} (99.3%)	~0.4 Ah cm^{-2}<br>~0.5 Ah cm^{-2}	3
LiFSI / TEP 1: 1.5 (molar ratio)	0.2 mA cm^{-2} / 1 mAh cm^{-2} (99.3%)	~0.7 Ah cm^{-2}	4
1.2 M LiFSI / DMC-BTFE (1:2 molar ratio)	0.5 mA cm^{-2} / 1 mAh cm^{-2} (99.0%)<br>5 mAh cm^{-2} Aurbach method (99.5%)	~0.4 Ah cm^{-2}<br>~0.04 Ah cm^{-2}	5
1.2 M LiFSI/ TEP-BTFE (1:3 molar ratio)	5 mAh cm^{-2} Aurbach method (99.2%)	~0.04 Ah cm^{-2}	6
1 M LiFSI/ DME-TFEO (1:3 molar ratio)	5 mAh cm^{-2} Aurbach method (99.5%)	~0.04 Ah cm^{-2}	7
1 M LiFSI / FDMB	0.25 mA cm^{-2}/0.5 mAh cm^{-2} (99.2%)<br>5 mAh cm^{-2} Aurbach method (99.52%)	~0.6 Ah cm^{-2}<br>~0.04 Ah cm^{-2}	8
1.3 M LiFSI + 0.3M THF/ FM-CO_2 (19:1 Weight ratio)	0.5 mA cm^{-2} / 0.5 mAh cm^{-2} (99.6%)	~0.5 Ah cm^{-2}	9
1.2 M LiFSI + 1M AN/ FM	3 mA cm^{-2} / 3 mAh cm^{-2} (99.4%)	~1.2 Ah cm^{-2}	10
0.5 M $LiNO_3$ + 2M LiFSI /DOL	1 mA cm^{-2} / **20 mAh cm^{-2} (99.33%)**	**~1.8 Ah cm^{-2}**	**This work**

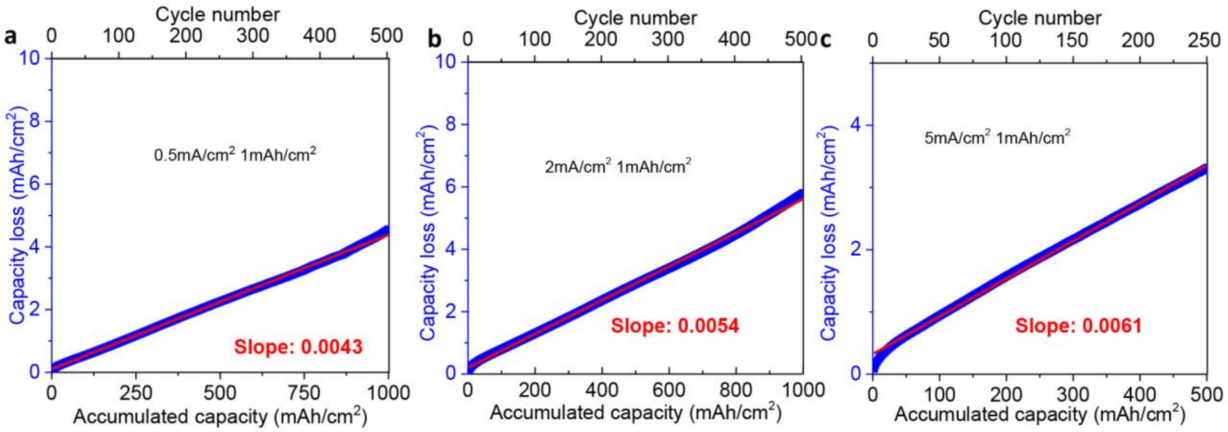

Supplementary Figure 12 | Lithium consumption rate at various current density. The current density of lithium stripping/plating is **a**, 0.5mA cm^{-2}, **b**, 2 mA cm^{-2} and **c**, 5 mA cm^{-2}. The plating capacity is 1 mAh cm^{-2}.

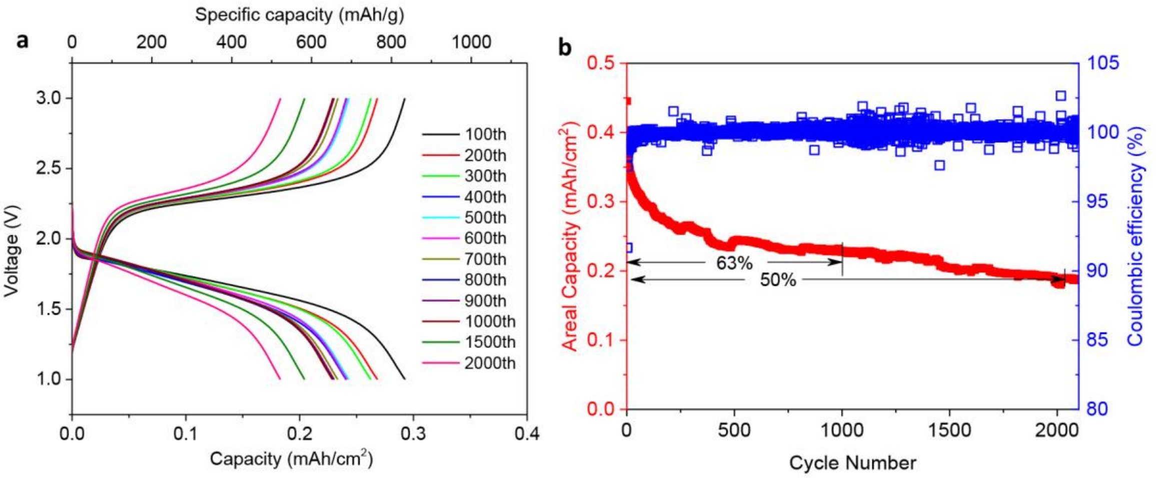

Supplementary Figure 13 | Li-S batteries with low areal capacity sulfur cathode and thin Li anode (50μm). **a**, Discharge/charge profiles and **b**, corresponding cycling performance with Coulombic efficiency. The current density for charge/discharge is 0.2 mA cm^{-2}.

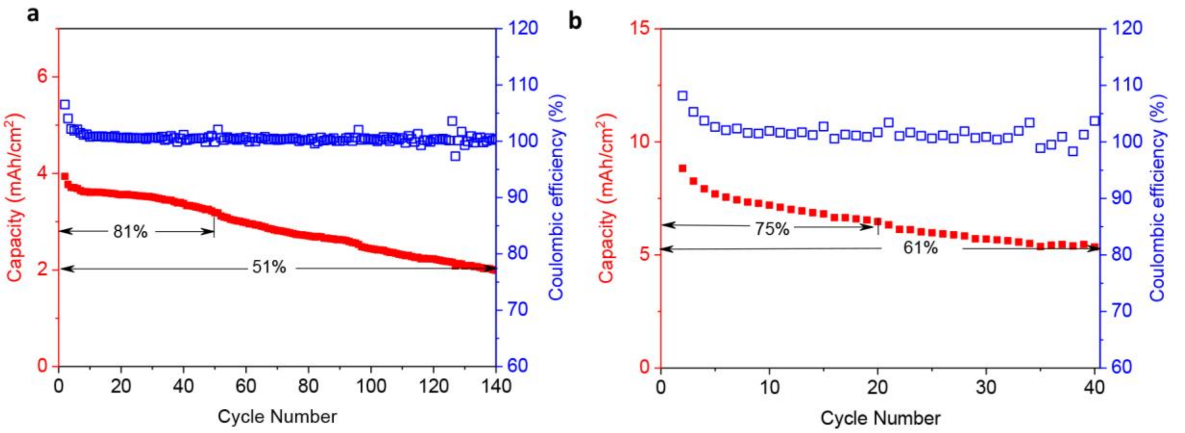

Supplementary Figure 14 | Li-S batteries with high areal capacity sulfur cathode and thin Li anode. **a**, **b** Cycling performance with Coulombic efficiency. This thickness of lithium is 50 μm for **a**, and 100 μm for **b**. The current density for charge/discharge is 0.5 mA cm^{-2}.

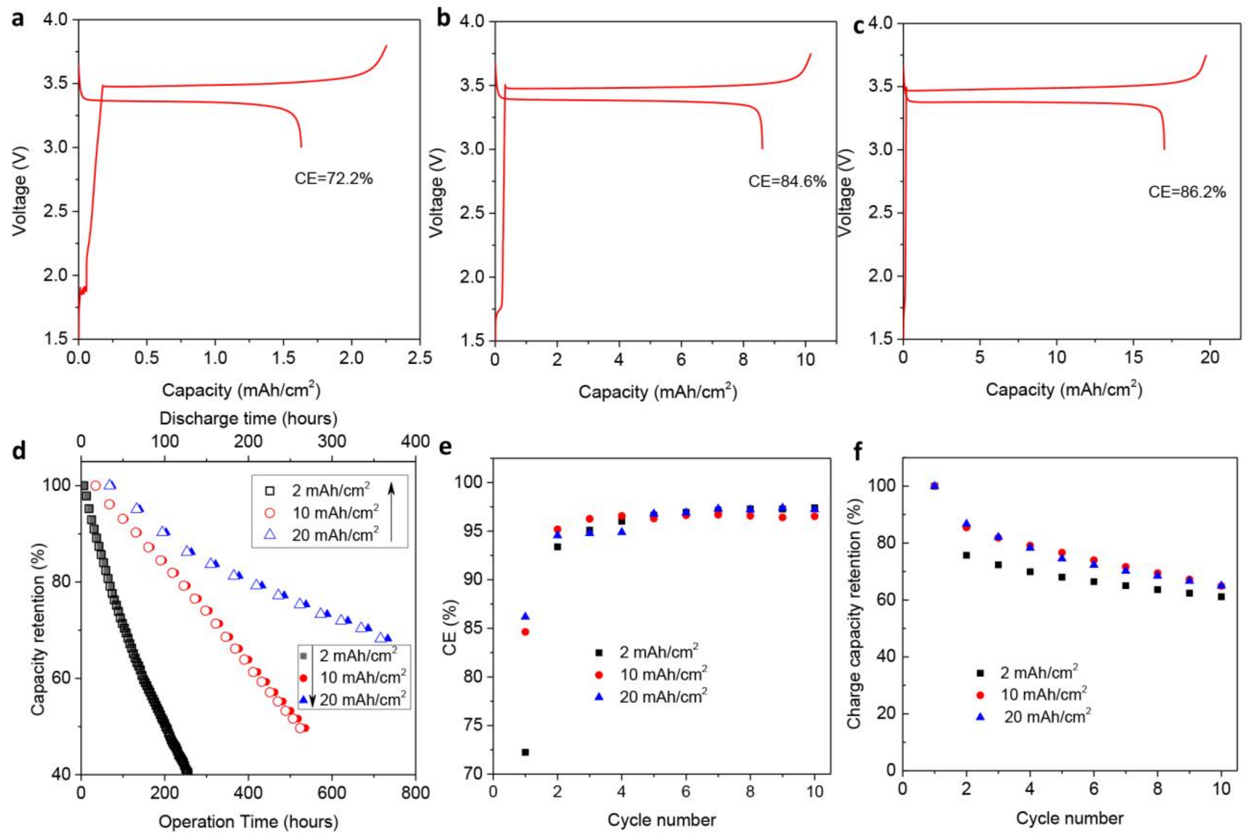

Supplementary Figure 15 | Anode-free Cu||LFP battery application. Initial charge/discharge profiles with designed areal capacity of **a,** 2 mAh cm^{-2}, **b,** 10 mAh cm^{-2}, and **c,** 20 mAh cm^{-2}. **d**, Capacity retention with operation time (Time of discharge + time of charge) or discharge time. **e,** Coulombic efficiency with cycle number. **f,** charge capacity retention with cycle number. The current density for charge and discharge is 0.5 mA cm^{-2}.

Note: The initial CE of Cu||LFP anode-free batteries increases with the increasing of areal capacity, confirming the higher Li reversibility at higher areal capacity. It should be mentioned that both DOL and LiFSI are not high voltage stable components, thus the CE is lower than 90% even for LFP with high mass loading.

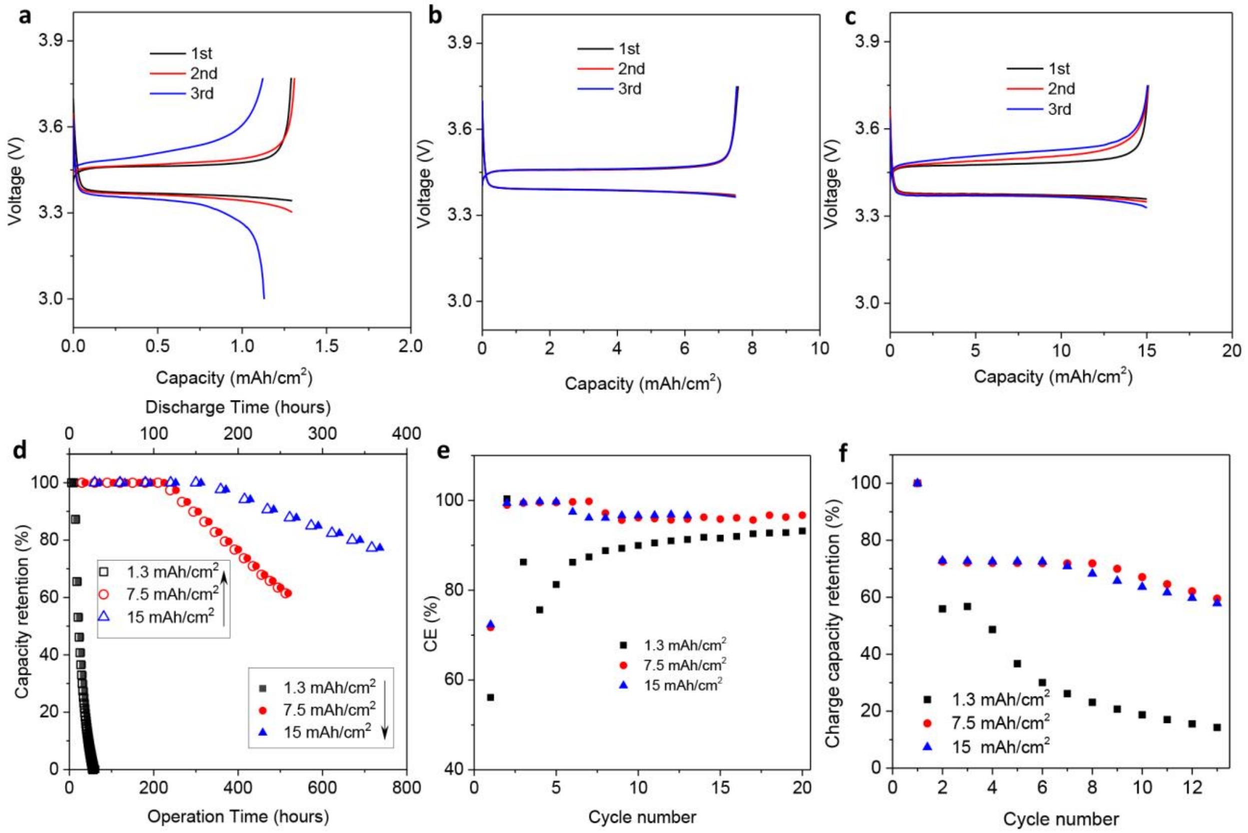

Supplementary Figure 16 | Cu||LFP battery applications with controlled discharge capacity. a-c, Charge/discharge profiles with designed theoretical areal capacity of **a,** 2 mAh cm^{-2}, **b,** 10 mAh cm^{-2}, and **c,** 20 mAh cm^{-2} and practically controlled discharge capacity of **a,** 1.3 mAh cm^{-2}, **b,** 7.5 mAh cm^{-2}, and **c,** 15 mAh cm^{-2}. **d,** Capacity retention with operation time (Time of discharge + time of charge) or discharge time. **e,** Coulombic efficiency with cycle number. **f,** Charge capacity retention with cycle number. The current density for charge and discharge is 0.5 mA cm^{-2}.

Note: Cu||LFP batteries with controlled discharge capacity are further designed to improve the long-term stability. LFP with same designed areal capacity of 2 mAh cm^{-2}, 10 mAh cm^{-2}, and 20 mAh cm^{-2} are used to assemble Cu||LFP batteries. The initial charge profiles are equal to Supplementary Figure 15. For the following discharge step, the process is stopped when the capacity reaches ~85% of theoretical capacity or the voltage decreases to 3.0 V. With small

portion of lithium remained on Cu, the cycling life are further prolonged with capacity retention over 80% after 700 hours.

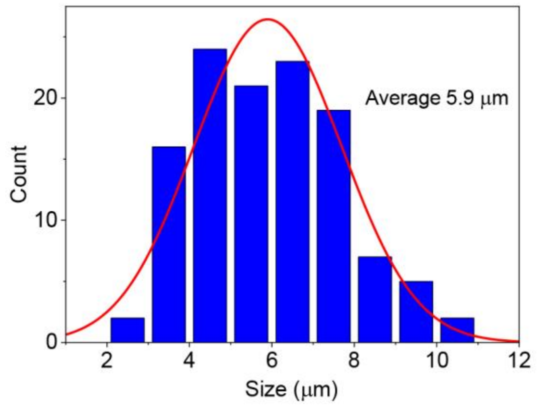

Supplementary Figure 17 | Pore size distributions of SEI on Cu substrate after removing deposited lithium (20 mAh cm^{-2}).

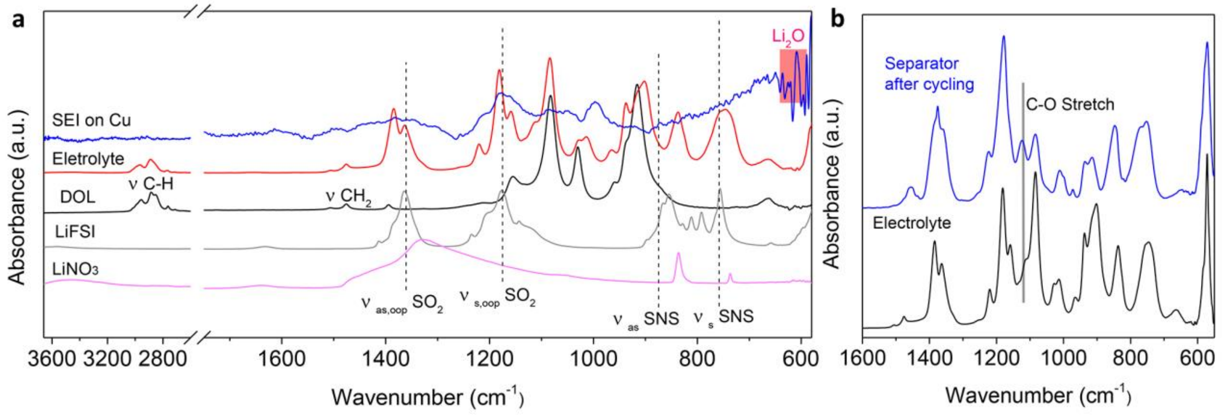

Supplementary Figure 18 | ATR-FTIR spectra of solid electrolyte interphase (SEI). a, insoluble SEI formed on Cu foil. **b**, Dynamic SEI capture by testing the separator after cycling.

Note: The disappearing of C-H vibration in left figure indicates the insoluble SEI on Cu is majorly inorganic compounds. In comparison, the soluble SEI can be found in the cycled electrolyte according to the new emerging peak of C-O stretch vibration.

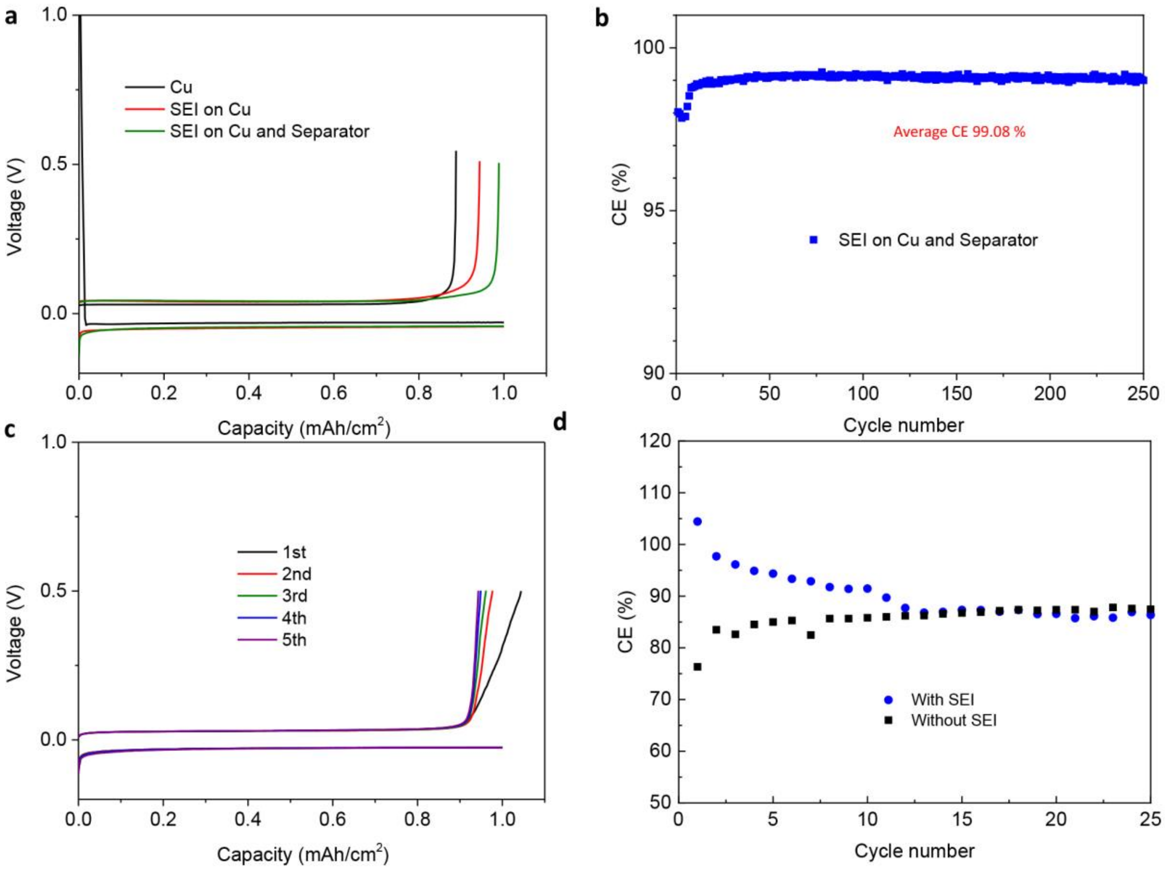

Supplementary Figure 19 | Effect of SEI on the reversibility of lithium anode. **a**, Initial lithium stripping/plating profiles and **b**, Coulombic efficiencies of Li||Cu electrochemical cells. The electrolyte in **a**, **b** is 0.5M $LiNO_3$ + 2M LiFSI in DOL. **c**, Lithium stripping/plating profiles with SEI protection and **d**, Coulombic efficiencies comparisons of Li||Cu electrochemical cells with or without the protection of SEI. The electrolyte in **c**, **d** is 1M $LiPF_6$ in EC/DMC.

References:

1 McCrory, C. C., Jung, S., Peters, J. C. & Jaramillo, T. F. Benchmarking heterogeneous electrocatalysts for the oxygen evolution reaction. *J. Am. Chem. Soc.***135**, 16977-16987 (2013).

2 Fu, J., Ji, X., Chen, J., Chen, L., Fan, X., Mu, D. & Wang, C. Lithium Nitrate Regulated Sulfone Electrolytes for Lithium Metal Batteries. Angew. Chem. Int. Ed. 59, 22194-22201 (2020).

3 Fan, X., Chen, L., Ji, X., Deng, T., Hou, S., Chen, J., Zheng, J., Wang, F., Jiang, J., Xu, K. & Wang, C. Highly Fluorinated Interphases Enable High-Voltage Li-Metal Batteries. *Chem* **4**, 174-185 (2018).

4 Xiao, L., Zeng, Z., Liu, X., Fang, Y., Jiang, X., Shao, Y., Zhuang, L., Ai, X., Yang, H., Cao, Y. & Liu, J. Stable Li Metal Anode with "Ion–Solvent-Coordinated" Nonflammable Electrolyte for Safe Li Metal Batteries. *ACS Energy Lett.* **4**, 483-488 (2019).

5 Chen, S., Zheng, J., Mei, D., Han, K. S., Engelhard, M. H., Zhao, W., Xu, W., Liu, J. & Zhang, J. G. High-Voltage Lithium-Metal Batteries Enabled by Localized High-Concentration Electrolytes. *Adv. Mater.* **30**, e1706102 (2018).

6 Chen, S., Zheng, J., Yu, L., Ren, X., Engelhard, M. H., Niu, C., Lee, H., Xu, W., Xiao, J., Liu, J. & Zhang, J.-G. High-Efficiency Lithium Metal Batteries with Fire-Retardant Electrolytes. *Joule*, **2,** 1548-1559 (2018).

7 Cao, X., Ren, X., Zou, L., Engelhard, M. H., Huang, W., Wang, H., Matthews, B. E., Lee, H., Niu, C., Arey, B. W., Cui, Y., Wang, C., Xiao, J., Liu, J., Xu, W. & Zhang, J.-G. Monolithic solid–electrolyte interphases formed in fluorinated orthoformate-based electrolytes minimize Li depletion and pulverization. *Nature Energy* **4**, 796-805 (2019).

8 Yu, Z., Wang, H. S., Kong, X., Huang, W., Tsao, Y. C., Mackanic, D. G., Wang, K. C., Wang,

X. C., Huang, W. X., Choudhury, S., Zheng, Y., Amanchukwu, C. V., Hung, S. T., Ma, Y. T., Lomeli, E. G., Qin, J., Cui, Y. & Bao, Z. N. Molecular design for electrolyte solvents enabling energy-dense and long-cycling lithium metal batteries. *Nature Energy* **5**, 526-533 (2020).

9 Yang, Y., Davies, D. M., Yin, Y., Borodin, O., Lee, J. Z., Fang, C., Olguin, M., Zhang, Y., Sablina, E. S., Wang, X., Rustomji, C. S. & Meng, Y. S. High-Efficiency Lithium-Metal Anode Enabled by Liquefied Gas Electrolytes. *Joule* **3**, 1986-2000 (2019).

10 Yang, Y., Yin, Y., Davies, D. M., Zhang, M., Mayer, M., Zhang, Y., Sablina, E. S., Wang, S., Lee, J. Z., Borodin, O., Rustomji, C. S. & Meng, Y. S. Liquefied gas electrolytes for wide-temperature lithium metal batteries. *Energy Environ. Sci.* **13**, 2209-2219 (2020).
